# Scanning in Diagnostics and Novel Solutions for the Protection of Built Heritage

**DOI:** 10.1155/2019/8190548

**Published:** 2019-11-15

**Authors:** Antonia Moropoulou, Elisabetta Zendri, Pilar Ortiz, George Fourlaris

**Affiliations:** ^1^Laboratory of Materials Science & Engineering, School of Chemical Engineering, National Technical University of Athens, 9 Iroon Polytechniou Str., Zografou Campus, Athens 15780, Greece; ^2^Department of Environmental Sciences, Informatics and Statistics, Ca' Foscari University of Venice, Mestre, Venice, Italy; ^3^Departamento de Sistemas Físicos, Químicos y Naturales, Universidad Pablo de Olavide, ES-41013 Seville, Spain; ^4^Laboratory of Physical Metallurgy, School of Mining and Metallurgical Engineering, National Technical University of Athens, 9 Iroon Polytechniou Str., Zografou Campus, Athens 15780, Greece

Protection of built heritage passes through three distinguished steps that involve the following:

- *Diagnosis*, that is, characterization of building materials and assessment of building materials' decay, as well as assessment of environmental loads;
- *Assessment* of compatibility and performance of conservation/restoration materials and interventions, both in lab and in situ on building scale;
- *Monitoring* of the structure after the application of the most appropriate materials and interventions, to assure sustainability.

Scanning microscopy techniques are among the techniques that are used throughout the investigation of the aforementioned processes, and their applicability is increasingly gaining ground. In this framework, the input aspects of the scanning microscopy techniques in the field of built heritage protection are of high importance. The enhancement of our knowledge on the physicochemical and microstructural characteristics of historic and modern building materials and their degradation processes, as well as on the properties of the conservation and repair materials that we need to design in terms of compatibility and sustainability, is vital, and scanning microscopy techniques are key tools towards this direction, with technological advances further increasing their effectiveness.

A. Moropoulou et al. review the literature for the use of scanning microscopy techniques in the fields of building materials and cultural heritage, comparing these techniques with other analytical techniques widely used in these fields, regarding acquired information, sample requirements, and major limitations. They also summarize the main advantages and limitations of the most commonly used scanning microscopy techniques in the field of built cultural heritage through numerically comparing the research papers that report their use and findings in bibliography and demonstrate that scanning electron microscopy with energy-dispersive X-ray spectroscopy analysis (SEM-EDX) can be regarded now as a routine and a key role technique, whereas transmission electron microscopy (TEM) is progressively utilized. Distinctive results of SEM-EDX are presented regarding building material characterization, such as stone and mortars; mortar production technology; stone decay diagnosis, as far as it concerns the impact of pollution, salt crystallisation, and biodeterioration; and assessment of cleaning, consolidation, and protection treatments. Furthermore, characteristic TEM results are displayed, revealing the presence of nanostructures in historical mortars, shedding light on their properties and their specific production technology. The use of TEM in the evaluation of the development of advanced nanomaterials, designed in particular for conservation treatments, such as consolidation and antifouling action, is also recorded.

M. Stefanidou and E. Pavlidou investigated the role of SEM-EDX in the characterization of historical mortars, not only in relation to their main components (binder, aggregates, and additives) but also in relation to their microstructure. It is demonstrated that important information about production technology of mortars and their preservation state can be acquired by SEM utilization. Furthermore, new repair mortars are to be designed with corresponding and compatible properties to the historical ones. In such cases, SEM results about raw material suitability, effectiveness evaluation of nanoparticle addition, and self-healing processes of special mortar additives are presented.

In the two following works of L. Ferrazza et al. and A. Gil-Torrano et al., the numerous ways that SEM-EDX can be coupled with several other techniques, in order to ensure the protection of built heritage, are demonstrated.

In particular, L. Ferrazza et al. focus on the conservation study of the facade of the Arciprestal Church of Santa María de Morella in Spain. A vast array of techniques such as ground penetration radar (GPR), X-ray fluorescence (XRF), optical microscopy (OM), X-ray diffraction (XRD), SEM-EDX, and Fourier transform infrared spectroscopy (FTIR) are used for building material characterization and decay diagnosis, while assessment of cleaning and consolidation treatments on architectural surfaces of both stones and polychromes is included. The multidisciplinary character of this work and the importance of SEM-EDX in investigating crusts, patina, and especially pigments and their substrates are highlighted.

Correspondingly, A. Gil-Torrano et al. investigate the archaeological site of Cercadilla in Cordoba, Spain, regarding the preservation state of wall paintings of the Roman era (3^rd^ to 4^th^ century AD) and the Caliphal era (10^th^ to 11^th^ century AD). The similarities and variances between the employed materials and technologies of the different eras' wall paintings are investigated through the combined use of OM, SEM-EDX, XRD and *μ*-XRD, wavelength-dispersive X-ray fluorescence (WD-XRF), and FTIR. The secco technique is used for the manufacturing of both wall paintings under investigation, while the wall paintings of the Roman era present more preparatory layers in comparison to the Arabic ones. Furthermore, red earth and calcite are detected as red and white pigments in the case of Roman paintings, whereas red ochre and calcite are identified as red and white pigments in the case of Arabic paintings.

On the other hand, G. Soupionis and L. Zoumpoulakis present the development of a heat resistant material that can be applied on modern built heritage structures to provide thermal insulation. In particular, they present the development of a new composite material, consisting of resite matrix, carbon fibers, and perlite, which aims at the protection of building envelopes during repair interventions. Different mix ratios of the composite's main components are investigated regarding their mechanical properties (flexural and shear strength) and heat resistance, whereas microstructure characteristics are examined by SEM-EDS.

Last but not least, S. Nicolopoulos et al. demonstrate a combination of ADT and ASTAR orientation/phase mapping techniques in TEM to investigate various materials with emphasis on pigments used in different artefacts, such as amphoriskos, glass tesserae, and Mayan mural paintings. Nanocrystalline mapping of pigments is achieved using the abovementioned TEM electron crystallography techniques. Ancient composite materials are thus thoroughly investigated, since chemical and structural information is acquired at nanoscale. Through this work, multiple scaled impacts are evident regarding the ways of how technological evolution can significantly enhance our knowledge in several areas of interest for the protection of cultural heritage.

The protection of built heritage is a vast and fascinating research field where many questions still have to be answered regarding the properties of building materials, as well as the weathering processes that govern their life cycle. Answering these questions will inspire scientists to pioneer in designing novel conservation/restoration and repair materials that will ensure structures' protection and sustainability.

In this context, scanning microscopy techniques play a key role in establishing methodologies and protocols towards revealing the past and planning the future. Technological advances further evolve the capabilities and the applicability of scanning microscopy techniques contributing to the sustainable protection of built heritage.

*Antonia Moropoulou* *Elisabetta Zendri* *Pilar Ortiz* *George Fourlaris*

